# Retraction: Intraoperative Cryoprecipitate Transfusion and Its Association with the Incidence of Biliary Complications after Liver Transplantation-A Retrospective Cohort Study

**DOI:** 10.1371/journal.pone.0222377

**Published:** 2019-09-06

**Authors:** The PLOS ONE Editors

Concerns have been raised that the transplants performed in the local context at the time of procedures reported in this article [1] may have involved organs/tissues procured from prisoners [2].

International ethics standards call for transparency in organ donor and transplantation programs and clear informed consent procedures including considerations to ensure that donors are not subject to coercion. Details as to the donor sources and methods of obtaining informed consent from donors were not reported in [1]. In response to the journal’s queries about these issues, the first author noted that the authors had access only to limited donor details (age, gender, blood type) in the clinical database used for this retrospective study, and that all transplants investigated in this study used organs/tissues from cardiac death donors that were 18 years or older. The authors did not provide information about donor consent, and the first author commented that the protocol for this study did not include details as to transplant donor sources or the procedure used to obtain informed consent from transplant donors. The authors did not provide documentation when requested by the journal to confirm that the study had institutional ethics approval.

The first author expressed that they stand by the results reported in this article but the individual-level data underlying the study’s results are no longer available.

Owing to the lack of documentation to demonstrate this study had prospective ethical approval, insufficient reporting, unresolved concerns around the source of transplanted organs and whether they included organs from prisoners, and in compliance with international ethical standards for organ/tissue donation and transplantation, the *PLOS ONE* Editors retract this article.

The authors did not respond to express whether they agree with the retraction decision.
